# Pseudo-Anterior ST-Elevation Myocardial Infarction Due to Hypertrophic Cardiomyopathy

**DOI:** 10.7759/cureus.97693

**Published:** 2025-11-24

**Authors:** Chukwuemeka Lekwa, Anwar H Thahakutty, Saad Ahmad, Nadeem Attar

**Affiliations:** 1 Cardiology, Blackpool Teaching Hospitals NHS Foundation Trust, Blackpool, GBR

**Keywords:** coronary artery angiography, hypertrophic cardiomyopathy (hcm), left ventricular hypertrophy (lvh), multimodality cardiac imaging, pseudo myocardial infarction

## Abstract

We report the case of a 43-year-old man with cardiovascular risk factors such as hypertension, hypertriglyceridemia and non-alcoholic fatty liver disease. He was admitted with a complaint of exertional and persistent chest pain, accompanied by electrocardiographic changes suggestive of anterior ST-elevation myocardial infarction. Initial investigations, including coronary angiography and optical coherence tomography, revealed unobstructed coronary arteries and no evidence of plaque disruption.

Transthoracic echocardiography demonstrated marked asymmetric septal hypertrophy with mild systolic anterior motion of the mitral valve and eccentric mitral regurgitation, in the absence of a resting or provoked left ventricular outflow tract gradient. Cardiac magnetic resonance imaging further confirmed a diagnosis of non-obstructive hypertrophic cardiomyopathy (HCM), with late gadolinium enhancement indicating patchy mid-septal and basal fibrosis.

In view of these findings, dual antiplatelet therapy was discontinued, and the patient was managed with beta-blockers. He was discharged with plans for outpatient cardiology follow-up, risk stratification for sudden cardiac death, and genetic evaluation.

This case highlights the potential for HCM to mimic acute coronary syndromes, particularly in a patient with cardiovascular risk factors, ischaemic ECG changes and ongoing chest pain. It underscores the diagnostic value of advanced imaging modalities in differentiating true myocardial infarction from phenocopies, thereby guiding appropriate and timely management.

## Introduction

Hypertrophic cardiomyopathy (HCM) is the most common inherited cardiac disorder, with an estimated prevalence of approximately 1 in 500 individuals worldwide. It was previously thought to cause pathogenic mutations of the sarcomere gene in an autosomal dominant manner, although many cases remain undiagnosed due to variable expression, incomplete penetrance, possible polygenic inheritance pattern and the contribution of non-genetic factors on disease phenotype. The condition is characterised by unexplained left ventricular hypertrophy (LVH), histologically defined by myocyte disarray, interstitial fibrosis, and small-vessel disease. Clinically, HCM is heterogeneous: many patients remain asymptomatic, while others present with exertional chest pain, dyspnoea, syncope, or arrhythmias, which may lead to sudden cardiac death. Current management of HCM is tailored to symptom control, risk stratification for sudden cardiac death, and prevention of disease progression, with therapies including beta-blockers, calcium channel blockers, septal reduction procedures, and implantable cardioverter-defibrillators in high-risk patients [1,2].

Importantly, myocardial ischaemia is a frequent and under-recognised feature of HCM, resulting not only from concomitant epicardial coronary artery disease but also from mechanisms such as coronary microvascular dysfunction (CMD), myocardial bridging, and increased oxygen demand from hypertrophied myocardium [3,4]. Although many patients remain asymptomatic, HCM can occasionally mimic acute coronary syndromes, particularly when chest pain and ST-segment elevation are present.

The capacity of HCM to imitate ST-elevation myocardial infarction (STEMI) represents an important diagnostic pitfall. This occurs because repolarisation abnormalities, microvascular ischaemia, and regional hypertrophy can produce ECG patterns that resemble true transmural infarction-raising the possibility of unnecessary thrombolysis, dual antiplatelet therapy, or urgent percutaneous coronary intervention. Such conditions are referred to as phenocopies, meaning disorders that appear similar to another disease but have a different underlying mechanism. Distinguishing true infarction from phenocopies often requires the use of multimodality imaging, which involves combining different cardiac imaging techniques, typically echocardiography, intracoronary imaging such as optical coherence tomography (OCT), and cardiac magnetic resonance imaging (CMR) to clarify the diagnosis. CMR frequently incorporates late gadolinium enhancement (LGE), a technique that highlights areas of myocardial scar or fibrosis by demonstrating delayed contrast washout compared with healthy myocardium.

Myocardial infarction with non-obstructive coronary arteries (MINOCA) is another important differential that requires consideration in such scenarios. Although the patient in this case did not meet the biomarker criterion for making a working diagnosis of MINOCA, several presenting features overlapped with defining features of the condition. A working diagnosis of MINOCA is usually made following angiography, but further investigations, including multimodality imaging and functional tests, are required to arrive at a final diagnosis and provide appropriate care. This framework is particularly valuable when evaluating ST-segment elevation with unobstructed coronary arteries, where distinguishing between MINOCA and structural heart disease such as HCM has major therapeutic implications.

Recognising these diagnostic challenges is critical, as patients with HCM and pseudo-STEMI may undergo unnecessary invasive procedures or receive long-term therapies intended for true myocardial infarction (MI). Multimodality imaging plays a pivotal role in distinguishing infarction from structural cardiomyopathies, myocarditis, microvascular disease, and MINOCA-related mechanisms, thereby preventing misdiagnosis and guiding disease-specific management.

We present this case because pseudo-anterior ST-segment elevation in the context of non-obstructive HCM is uncommon and may closely resemble anterior STEMI or MINOCA on initial evaluation. This case highlights the diagnostic value of integrating echocardiography, OCT, and CMR early in the assessment of patients with ST-segment elevation and unobstructed coronary arteries, emphasising the importance of recognising HCM as a potential phenocopy to avoid unnecessary intervention and ensure appropriate long-term management.

An abstract of this case report was presented as a digital poster at the 2025 Royal College of Physicians and Surgeons of Glasgow Interactive Cardiology Conference on March 7, 2025.

## Case presentation

A 43-year-old man was admitted with a three-week history of exertional and ongoing chest pain as his sole presenting symptom. The chest pain was described as a sensation of heaviness, non-radiating and without identifiable relieving factors. His medical history included hypertension, hypertriglyceridemia, non-alcoholic fatty liver disease, Class 1 obesity with a body mass index of 34.6 kg/m^2^, and a family history of ischaemic heart disease and HCM.

**Figure 1:**
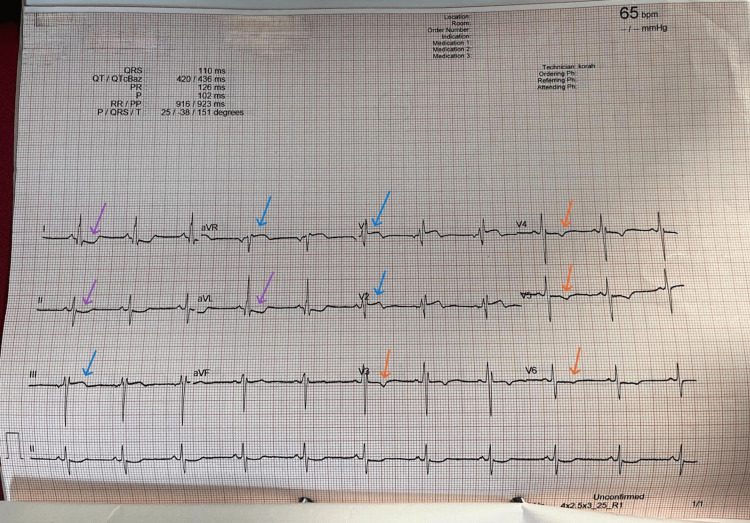
ECG at presentation 12-lead electrocardiogram (ECG): Sinus rhythm with ST elevation in aVR, V1-V2, and lead III (blue arrows), T wave inversions V3-V6 (orange arrows), and ST depression in leads I, II and aVL (purple arrows). The V1-V2/aVR elevation and widespread T wave inversion can be seen in HCM, whereas inferior involvement is more typical of myocardial infarction. HCM: Hypertrophic cardiomyopathy

On admission, his ECG showed sinus rhythm with left axis deviation, incomplete right bundle branch block, ST elevation in V1-V2, aVR, and lead III, ST depression in leads I, II, aVL, and T wave inversion in V3-V6 (Figure 1).

No prior ECG was available for comparison. Serial troponin levels performed seven and a half hours apart were 39 ng/L and 33 ng/L, respectively (normal range: 0-47 ng/L, downward trend suggesting no ongoing myocardial injury). The calculated HEART score was 4, consistent with an intermediate risk for major adverse cardiac events.

Routine laboratory tests, including HbA1c (40 mmol/mol), cholesterol, liver, and renal function (estimated glomerular filtration rate >90 mL/min/1.73 m²), were all within the normal reference ranges. All laboratory results and BMI are summarised in Table 1. 

**Table 1:** Laboratory and clinical results on admission BMI: body mass index; HbA1c: haemoglobin A1c; GFR: glomerular filtration rate; ALP: alkaline phosphatase; ALT: alanine aminotransferase.

Test	Result	Reference Range	Units
Body mass index (BMI)	34.6	18.5 – 24.9	kg/m²
High-sensitivity troponin	39 → 33	0 – 47	ng/L
Haemoglobin A1c (HbA1c)	40	20 – 41	mmol/mol
Estimated GFR	>90	>90 (normal)	mL/min/1.73 m²
Albumin	39	35 – 50	g/L
Bilirubin	9	0 – 20	µmol/L
Alkaline phosphatase (ALP)	69	30 – 130	IU/L
Alanine aminotransferase (ALT)	35	0 – 49	IU/L
Urea	4.1	2.5 – 7.8	mmol/L
Creatinine	78	64 – 104	µmol/L
Sodium	141	133 – 146	mmol/L
Potassium	4.5	3.5 – 5.3	mmol/L
Cholesterol	4.4	0 – 5	mmol/L

**Figure 2:**
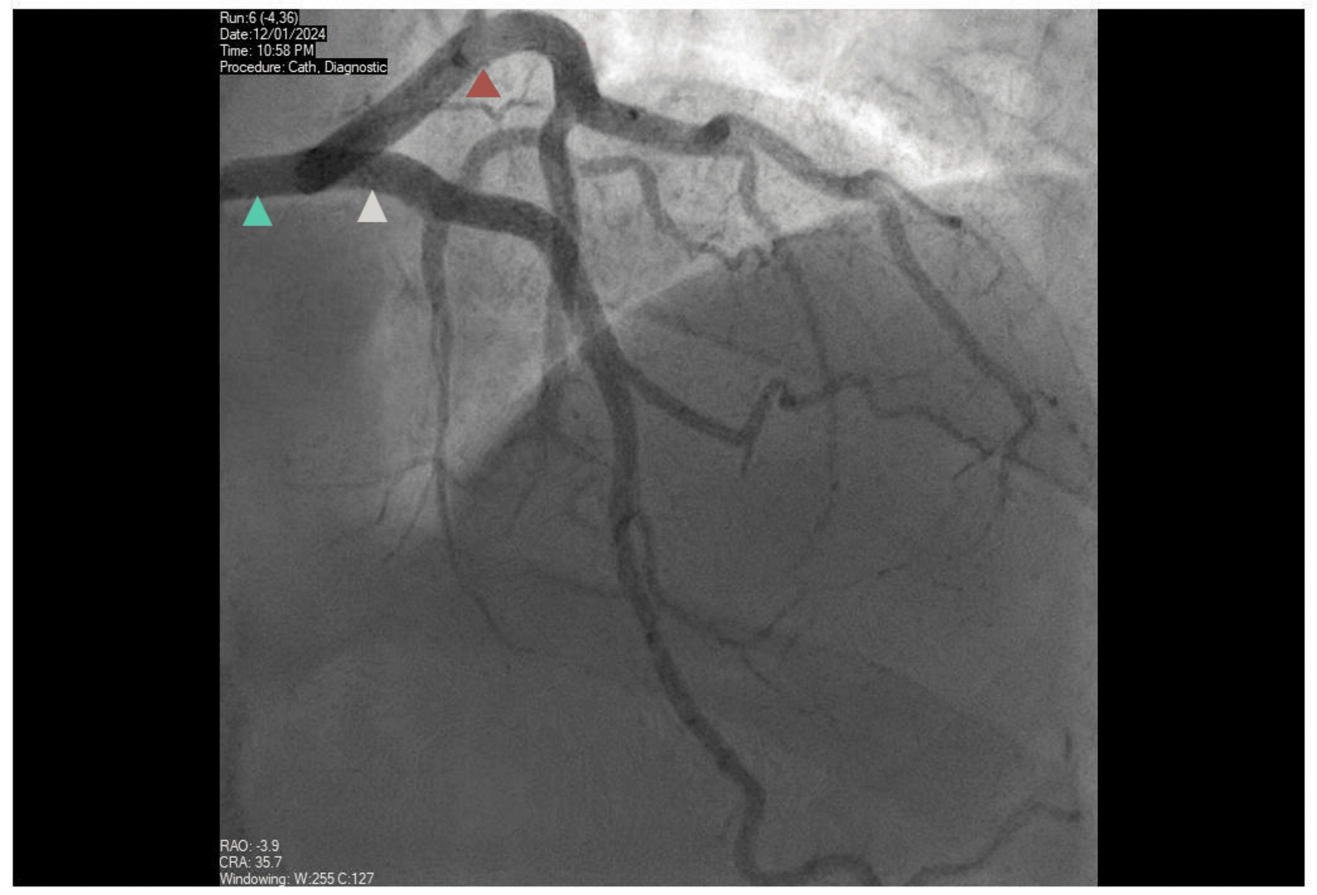
Coronary angiography - left coronary arteries Unobstructed left main stem (green arrow), left anterior descending (yellow arrow), and circumflex (orange arrow) arteries.

**Figure 3:**
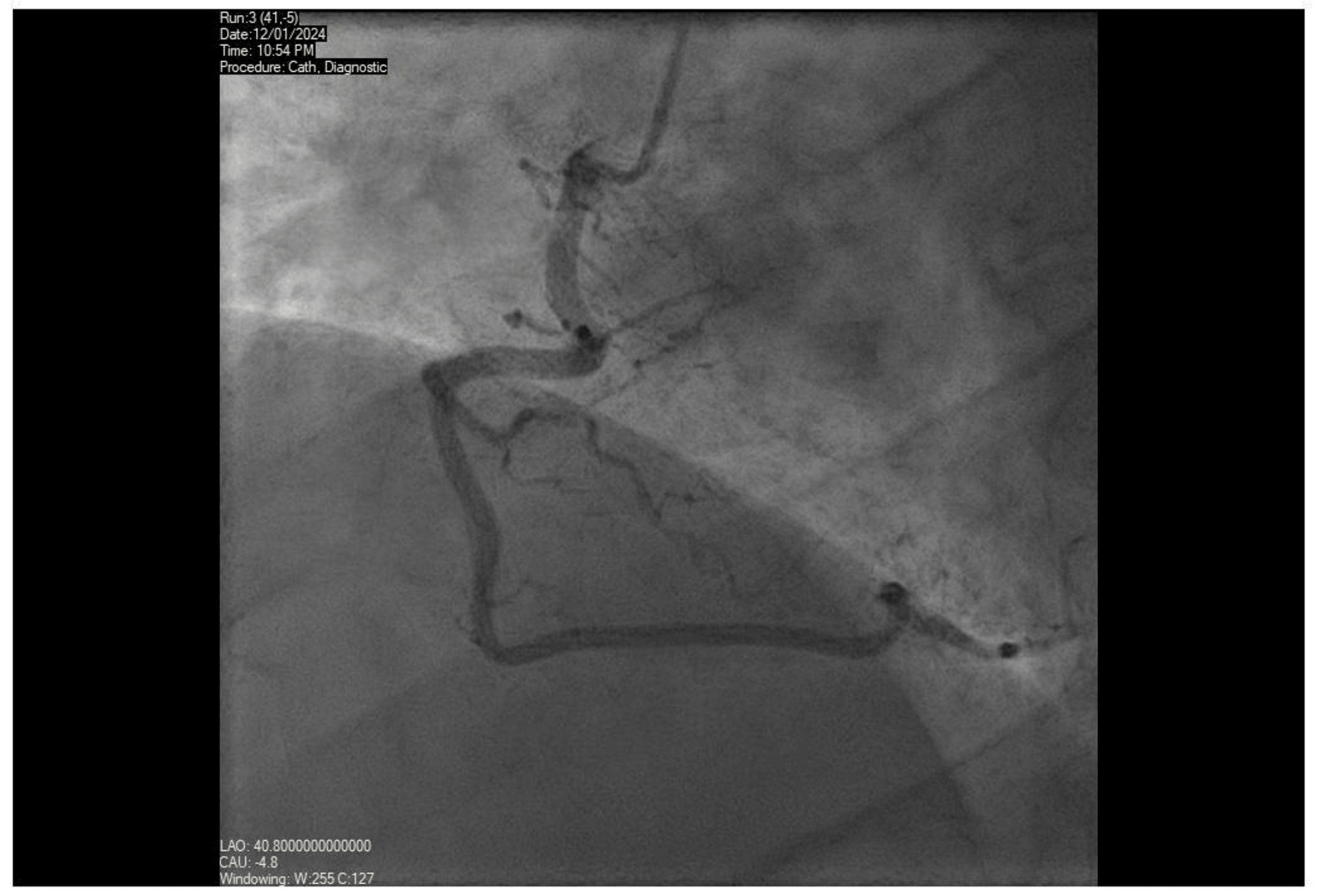
Coronary angiography - right coronary artery Unobstructed right coronary artery.

Loading doses of aspirin 300mg, ticagrelor 180mg were administered and he underwent invasive coronary angiography, also receiving intravenous unfractionated heparin (five thousand units) during the procedure. Coronary angiography demonstrated unobstructed coronary arteries (Figures 2, 3).

OCT was then performed and demonstrated a smooth, uniformly layered vessel wall without abnormalities. No rupture, thrombus, or significant plaque was seen to account for the presentation (Video 1).

**Video 1:** Optical coherence tomography of the left anterior descending artery Intravascular imaging clip showing circumferential and well-apposed arterial lumen with intact three-layered architecture and no evidence of plaque rupture, thrombus, or significant atheroma.

**Figure 4:**
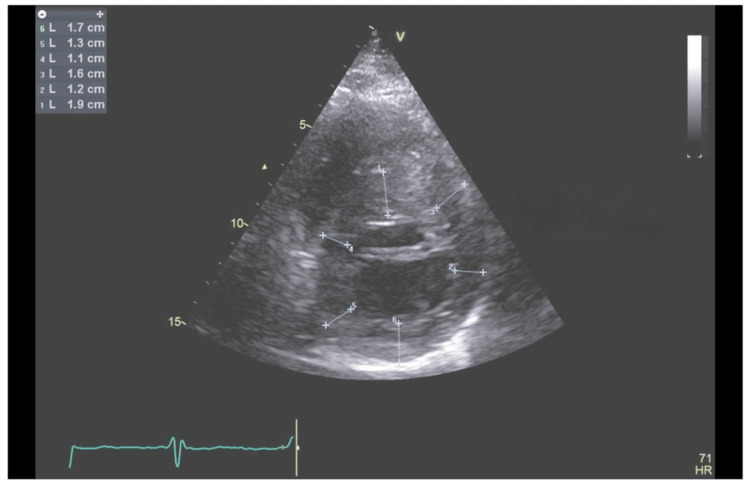
Echocardiogram - parasternal short-axis view with wall thickness measurements Multiple measurements showing septal thickness ranging from 1.1 to 1.9 centimetres confirming septal hypertrophy.

**Figure 5:**
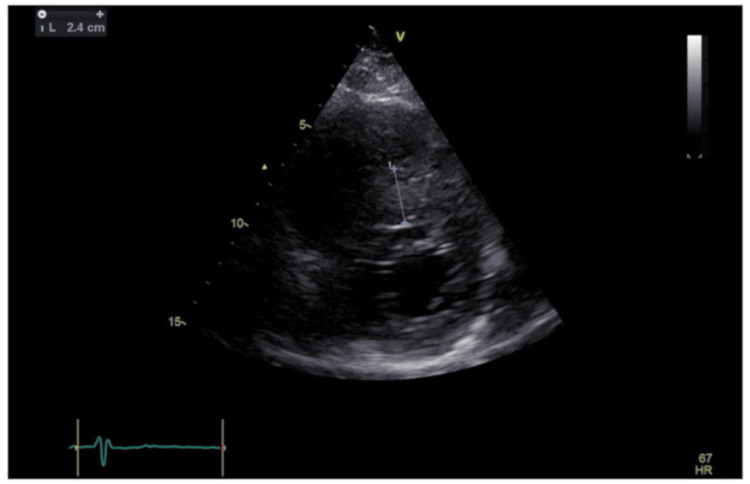
Echocardiogram- parasternal short-axis at the apical level Marked asymmetric septal hypertrophy measuring 2.4 centimetres.

**Figure 6:**
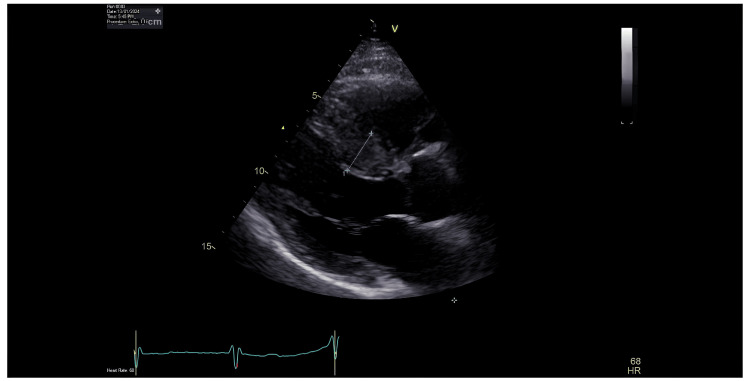
Echocardiogram - parasternal long-axis view Pronounced asymmetric septal hypertrophy.

As invasive coronary angiography and intravascular imaging failed to explain the patient’s presenting symptoms and ECG changes, further imaging with transthoracic echocardiography and cardiac magnetic resonance imaging was undertaken. Transthoracic echocardiography demonstrated severe asymmetric septal hypertrophy with a maximum wall thickness of 24 mm, minor systolic anterior motion of the mitral valve, and mild eccentric mitral regurgitation (Figures 4-6).

Left ventricular outflow tract (LVOT) gradients were 5 mmHg at rest and 6 mmHg on Valsalva, confirming the absence of obstruction, which is typically defined as a peak gradient of ≥30 mmHg. Left ventricular systolic function and left atrial dimensions were normal, with no regional wall motion abnormalities.

**Figure 7:**
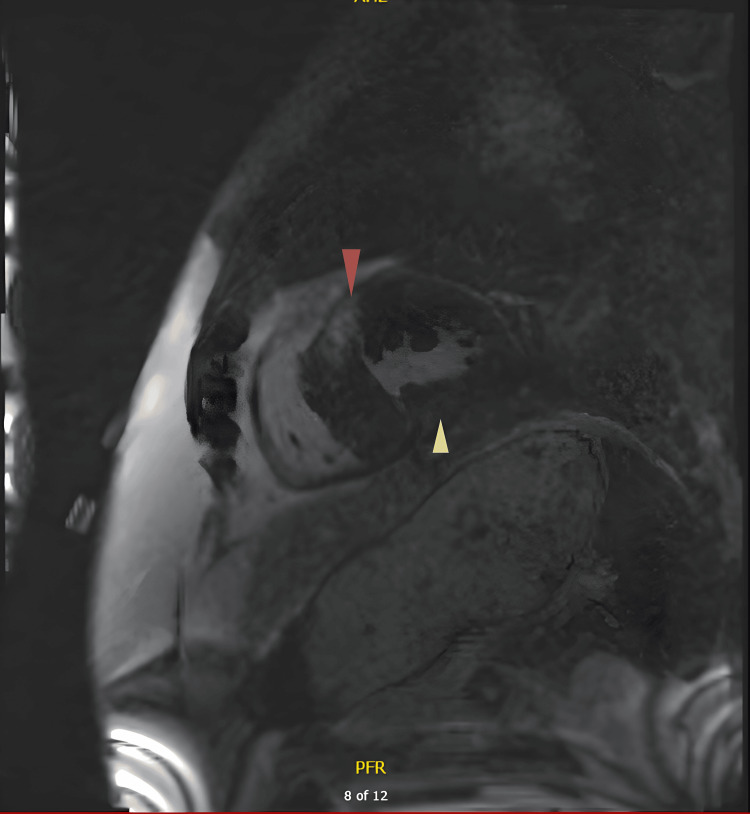
Cardiac magnetic resonance imaging - short-axis view at the papillary muscle level Patchy mid-wall late gadolinium enhancement (red and yellow arrows) at mid anteroseptum and inferoseptum, indicating myocardial fibrosis.

**Figure 8:**
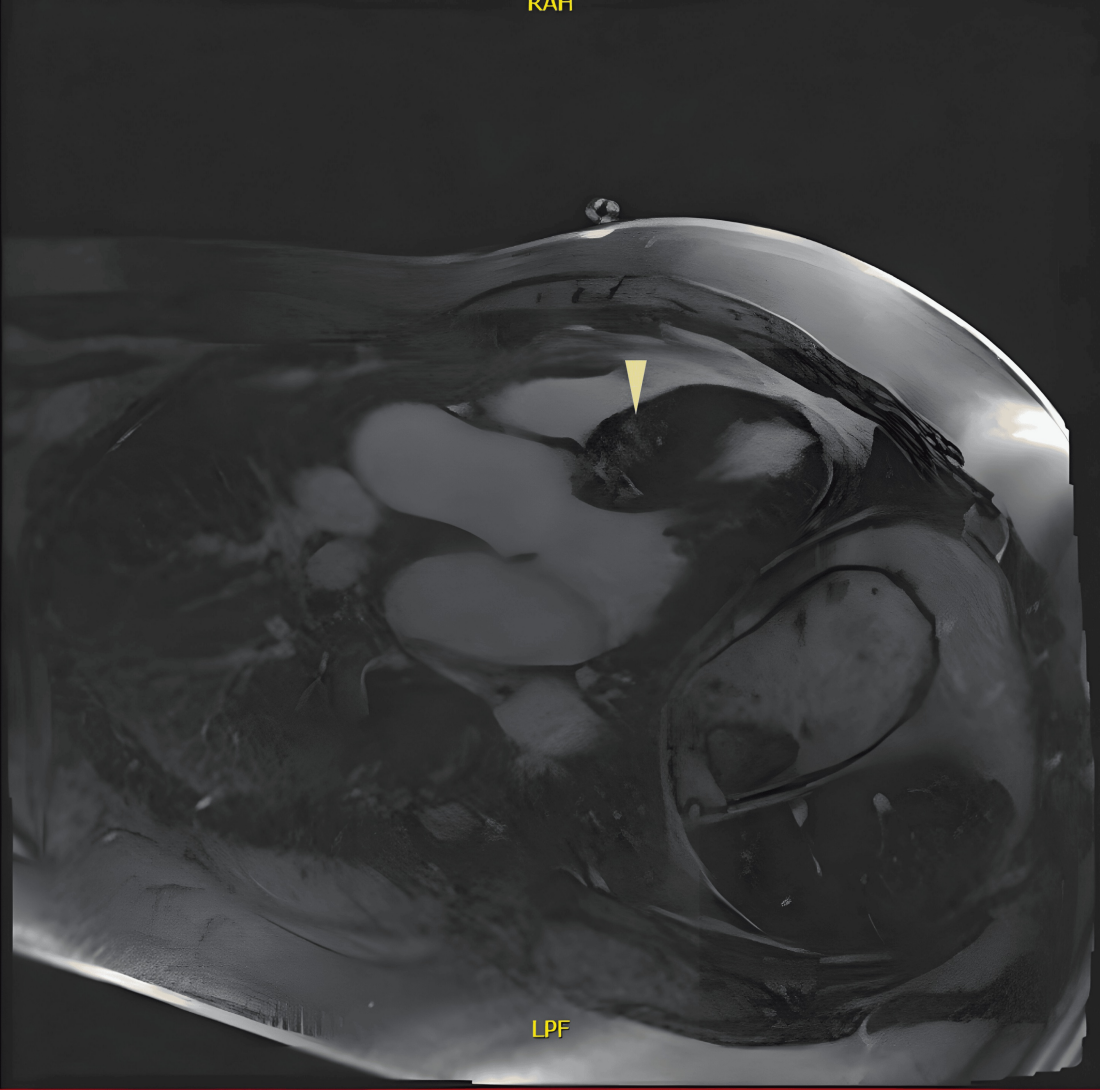
Cardiac magnetic resonance imaging - three-chamber view Extensive mid-wall late gadolinium enhancement (yellow arrow) involving basal to mid anteroseptum.

Cardiac MRI confirmed non-obstructive HCM predominantly affecting the basal and mid-ventricular septum. LGE indicated patchy fibrosis in these regions, along with mild bi-atrial dilatation and preserved left ventricular systolic function at 60% (Figures 7, 8).

**Figure 9:**
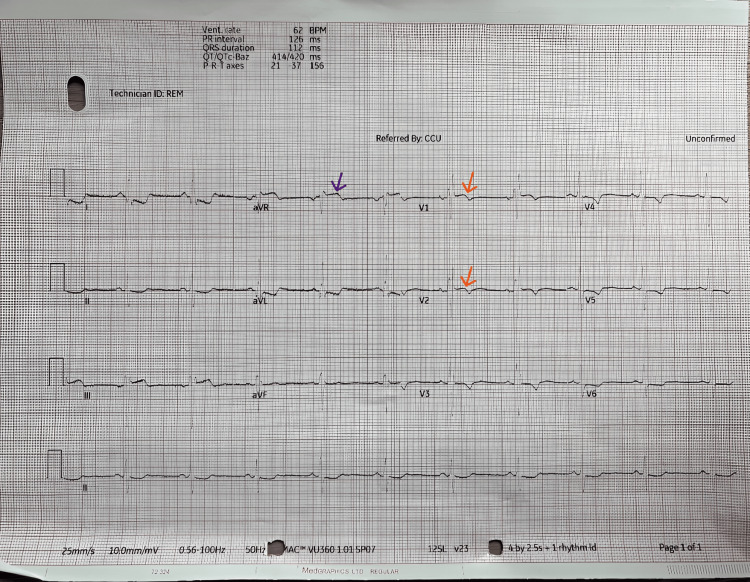
Electrocardiogram at discharge Resolution of ST elevation in leads V1-V2 (orange arrows) and persistent ST elevation in aVR (purple arrow), a pattern that may be seen in both hypertrophic cardiomyopathy and ischaemic or non-ischaemic conditions, requiring correlation with clinical and imaging findings.

Considering these findings, dual antiplatelet therapy was discontinued. He was then started on bisoprolol 2.5mg daily in addition to his preadmission ramipril 5mg daily and discharged with plans for cardiology follow-up to monitor disease progression, evaluate risk for sudden cardiac death, and initiate genetic testing. Serial ECGs showed resolution of the ST-segment elevations in V1-V2, although the elevation in aVR persisted at discharge (Figure 9).

## Discussion

This case illustrates the diagnostic challenges of differentiating true MI from mimics such as HCM. HCM is characterised by LVH that is unexplained by abnormal loading conditions, myocyte hypertrophy, and disarray, and increased myocardial fibrosis. While many patients remain asymptomatic, symptoms, when present, result from diastolic left ventricular dysfunction, LVOT obstruction, myocardial oxygen demand and supply imbalance, cardiac arrhythmias and sudden cardiac death [5].

Myocardial ischaemia is common in HCM and is associated with heart failure, malignant arrhythmias, and sudden cardiac death. The mechanism of ischaemia in HCM may be varied; however, CMD is considered the principal mechanism in the absence of epicardial disease. CMD contributes to progressive myocardial fibrosis and eventual myocyte death. Ischaemia may also occur due to co-existing epicardial coronary artery disease or may result from increased oxygen demand from the larger myocardial mass, elevated filling pressures, anomalous coronaries, and myocardial bridging, causing ischaemia by prolonged coronary compression and reduced blood flow in the early diastolic phase [4].

Typical ECG findings in HCM include pathological Q waves with an upright T-wave in the same leads, usually due to abnormal septal hypertrophy. In apical variants, giant symmetric T-wave inversions are noted rather than Q waves. Other findings include deep S-waves in V1-V3, or high R-waves in V4-V6 due to LVH with abnormal T-waves. Mild ST-T wave modifications, lonely diphasic T-waves, isolated inverted T-wave in aVL may sometimes be the only abnormality. The ECG in HCM may also look normal in 4 to 6% of adult patients and less than 3% in paediatric cases. Prominent ST-segment elevation (pseudo-STEMI pattern) in anterior leads is not rare and was identified in 17% of patients [6]. However, Gupta et al. reported that among nearly six million patients presenting with acute MI, only 0.1% had HCM, and those individuals were more likely to have non-ST elevation MI than STEMI [7]. Potential mechanisms for ST-segment elevation in HCM include apical aneurysm (notably in leads V4-V6) [8], repolarisation changes from LVH (especially in leads V1-V3) [9], and myocardial fibrosis with myocyte disarray that impairs depolarisation of local myocardium and generates locally oriented ST vectors. Additionally, abnormal polarisation of hypertrophied myocytes can produce an injury current similar to that seen in acute MI [10].

ST elevation in acute MI is defined electrocardiographically as new ST-elevation at the J-point in ≥ 2 contiguous leads, ≥2.5 mm in men < 40 years, ≥ 2mm in men ≥40 years, or ≥1.5 mm in women regardless of age in leads V2−V3 and/or ≥1 mm in the other leads (in the absence of LV hypertrophy or left bundle branch block) [11]. In HCM, STE may be persistent or dynamic and is often unaccompanied by reciprocal ST depression, which is common in STEMI [10,12-16]. Several ECG clues may help distinguish ST-segment elevation in HCM from acute MI. The presence of reciprocal ST-segment depression and serial ECGs demonstrating the dynamic nature of the ST-segment/T-wave abnormalities over very short periods of time favours acute MI [17]. 

Yang et al. demonstrated that pseudo-STEMI patterns in HCM typically involve fewer leads and lower overall ST-segment elevation than true STEMI, with STE most often localised to V1-V2 and aVR due to septal hypertrophy and basal septal involvement. They also reported that reciprocal ST depression, common in acute infarction, was infrequent and diagnostically weak in HCM, while widespread and persistent T-wave inversion, especially in lateral and inferior leads, was far more characteristic. Importantly, abnormal Q waves were not reliable discriminators, as they may arise from fibrosis or asymmetric hypertrophy rather than infarction [18]. These findings reinforce that ECG alone cannot distinguish HCM from STEMI and highlight the need for multimodality imaging, as in this case, where normal serial high-sensitivity troponin values (39 ng/L falling to 33 ng/L) and unobstructed coronaries supported the absence of MI. Of particular relevance to this patient, the persistent STE in lead aVR despite resolution of anterior STE parallels the observation by Yang et al. that aVR changes may be misleading and require imaging correlation rather than assumptions of ischaemia [18].

It is also important to consider other differential diagnoses when ST-segment elevation is seen on the ECG. These include but are not limited to pericarditis/myopericarditis, typically producing diffuse concave STE with PR-segment depression; early repolarisation, often seen in young adults with concave upward ST-segment elevation typically widespread in the precordial (and sometimes limb) leads, often with slurring or notching at the J point, and prominent concordant asymmetric T waves; left ventricular aneurysm, which produces persistent STE in the context of previous acute MI and abnormal Q waves; and Brugada syndrome, characterized by coved or saddleback STE in V1-V2 with complete or incomplete right bundle branch block [17]. Takotsubo cardiomyopathy, which can mimic anterior STEMI by showing ST elevation, isolated T-wave inversion, or no abnormalities, but can be distinguished by lower precordial ST elevation, absence of Q waves, and no changes in V1 or aVR [19].

Another clinically important differential to consider is MINOCA, an umbrella term encompassing a heterogeneous range of coronary and non-coronary causes. Although MINOCA most commonly presents as a non-ST-elevation myocardial infarction, occurring in approximately 70-80% of cases, it can occasionally present with ST-segment elevation and therefore warrants consideration in atypical presentations [20]. It is a working diagnosis that is made when a patient presents with symptoms suggestive of acute coronary syndrome, demonstrates troponin elevation and less than fifty percent stenosis in any major epicardial vessel at the time of angiography. Further assessment, including measurement of microvascular function/coronary reactivity, intravascular and non-invasive imaging, is required to determine the underlying aetiology [11]. Diagnostic yield increases when different imaging modalities are combined. In the HARP study, a combination of OCT and CMRI enabled diagnosis in 85% of cases, compared to just 46% with OCT alone and 74% with CMRI alone [20]. Although our patient shared some features of MINOCA such as ischaemic sounding chest pain and ECG changes, troponin levels did not exceed the 99th percentile of the normal limit.

Multimodal imaging, especially echocardiography and cardiac MRI with late gadolinium enhancement, is essential for establishing the correct diagnosis and avoiding unwarranted long-term antiplatelet therapy or revascularisation procedures [21].

## Conclusions

This case demonstrates that persistent anterior ST-segment elevation in the absence of obstructive coronary disease should prompt consideration of hypertrophic cardiomyopathy as a key STEMI mimic. Multimodality imaging, particularly bedside echocardiography and cardiac MRI with LGE, plays a critical role in rapidly distinguishing true infarction from phenocopies, preventing unnecessary dual antiplatelet therapy or revascularisation. Incorporating early access to echocardiography into STEMI triage pathways may help reduce inappropriate primary percutaneous coronary intervention activations and improve diagnostic accuracy in similar presentations. Of note, the patient remained clinically stable on the medical therapy initiated during his index admission when reviewed by his local cardiology team at follow-up. This favourable progress further supports the appropriateness of the management approach adopted.
